# Supplementary material for: Crosstalk between SUMOylation and ubiquitylation controls DNA end resection by maintaining MRE11 homeostasis on chromatin
Source: Nat Commun. 2022 Sep 1;13:5133. doi: 10.1038/s41467-022-32920-x (PMC9436968; doi:10.1038/s41467-022-32920-x)
Supplement: Supplementary file 1 — Supplementary Information [file 41467_2022_32920_MOESM1_ESM.pdf]

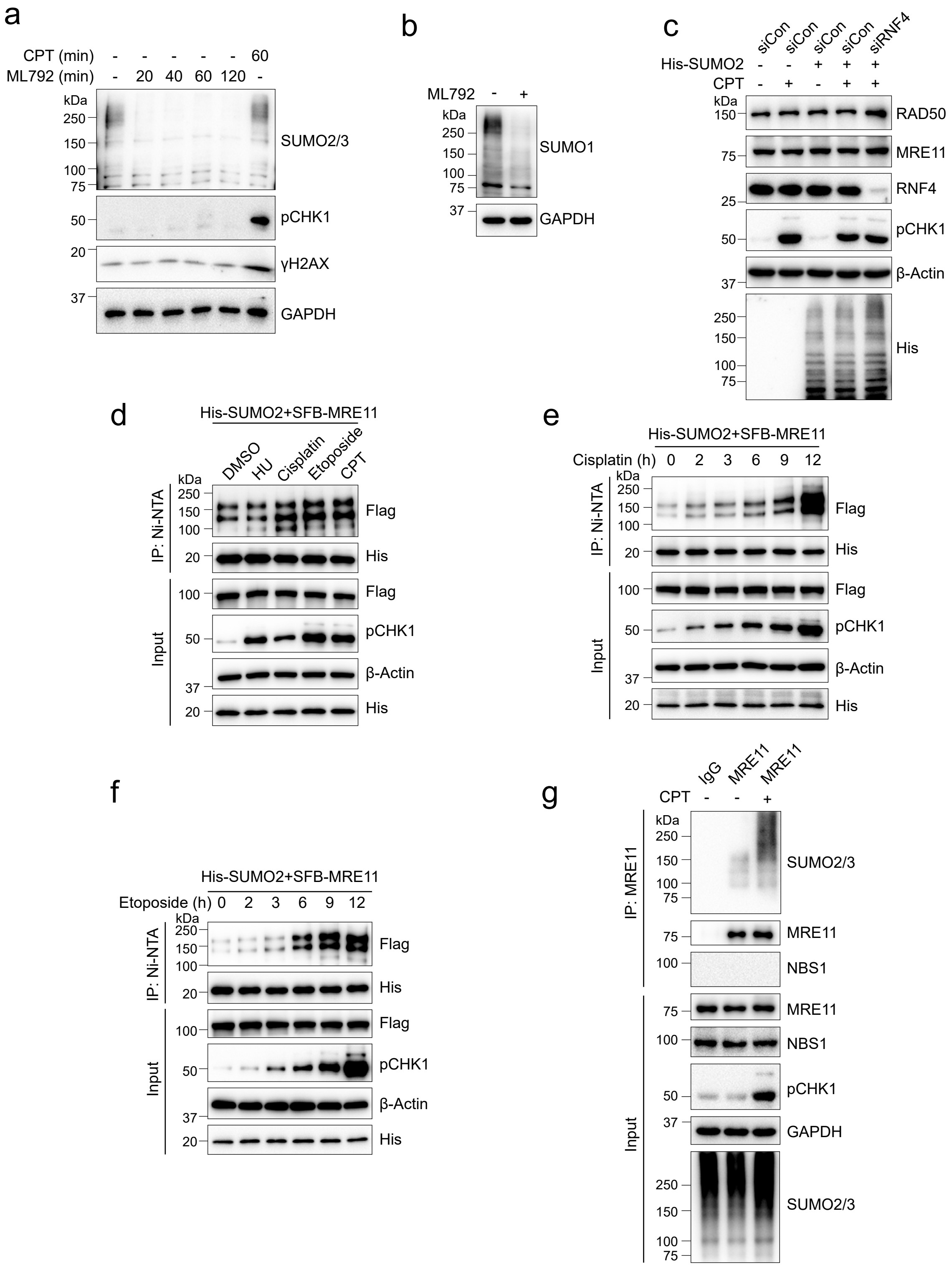

a
CPT (min) - - - - - 60
ML792 (min) - 20 40 60 120 -
kDa
250
150
100
75
SUMO2/3
50
pCHK1
20
γH2AX
37
GAPDH
b
ML792 - +
kDa
250
150
100
75
SUMO1
37
GAPDH
c
siCon siCon siCon siCon siRNF4
His-SUMO2 - - + + +
CPT - + - + +
kDa
150
RAD50
75
MRE11
25
RNF4
50
pCHK1
37
β-Actin
250
150
100
75
His
d
His-SUMO2+SFB-MRE11
DMSO HU Cisplatin Etoposide CPT
kDa
250
150
100
Flag
20
His
IP: Ni-NTA
100
Flag
50
pCHK1
37
β-Actin
20
His
Input
e
His-SUMO2+SFB-MRE11
Cisplatin (h) 0 2 3 6 9 12
kDa
250
150
100
Flag
20
His
IP: Ni-NTA
100
Flag
50
pCHK1
37
β-Actin
20
His
Input
f
His-SUMO2+SFB-MRE11
Etoposide (h) 0 2 3 6 9 12
kDa
250
150
100
Flag
20
His
IP: Ni-NTA
100
Flag
50
pCHK1
37
β-Actin
20
His
Input
g
IgG MRE11 MRE11
CPT - - +
kDa
250
150
100
75
SUMO2/3
75
MRE11
100
NBS1
IP: MRE11
75
MRE11
100
NBS1
50
pCHK1
37
GAPDH
250
150
100
75
SUMO2/3
Input

Supplementary Fig. 1 MRE11 SUMOylation is increased in response to DNA damage.

a SUMO E1 inhibitor ML792 (20 μM) treatment for the indicated times did not cause DNA damage. 1 h of 1 μM CPT treatment was used as a positive DNA damage control. **b** ML792 inhibits SUMO1-mediated SUMOylation in whole cell extracts. **c** RAD50 protein level was enhanced by RNF4 knockdown in HeLa cells expressing His-SUMO2 after CPT treatment. **d** HeLa cells expressing SFB-MRE11 and His-SUMO2 were treated with DNA-damaging agents (1 μM CPT for 2 h, 100 μM etoposide for 3 h, 1 μM cisplatin for 3 h, and 2 mM HU for 3 h). Then, MRE11 SUMOylation was analyzed by denaturing pull-down and immunoblotting. **e** and **f** SUMOylation of MRE11 was enhanced after cisplatin and etoposide treatment for the indicated times. **g** Endogenous MRE11 SUMOylated by endogenous SUMO2/3 was detected in HEK293T treated with or without CPT (1 μM, 8 h). Source data are provided as a Source Data file.

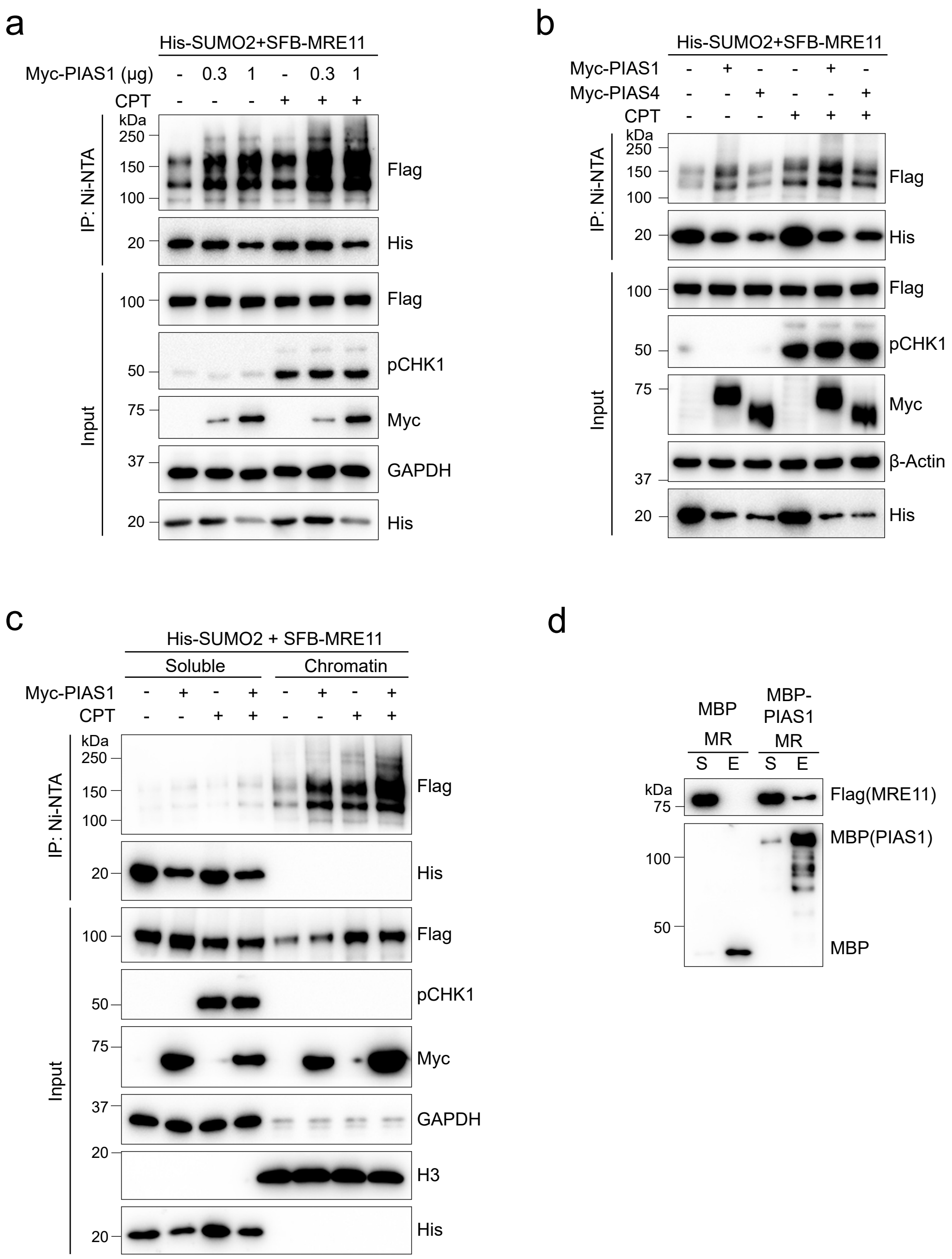

Supplementary Fig. 2 PIAS1 is the major SUMO E3 ligase for MRE11.

a MRE11 SUMOylation in cells with increasing levels of Myc-PIAS1 was analyzed by Ni-NTA pull-down and immunoblotting. **b** HEK293T cells co-transfected with His-SUMO2, SFB-MRE11 and Myc-PIAS1/PIAS4 were treated with 1 μM CPT for 8 h or not. Then, SUMOylation of MRE11 was examined by immunoblotting. **c** HEK293T cells expressing the indicated plasmids were fractionated into soluble and chromatin fractions, followed by immunoblotting for MRE11 SUMOylation. **d** Purified MBP-PIAS1 (150 nM) and MR (50 nM) were incubated and pulled down by amylose beads, and the supernatant (S) and the eluate (E) were subjected to immunoblotting. Source data are provided as a Source Data file.

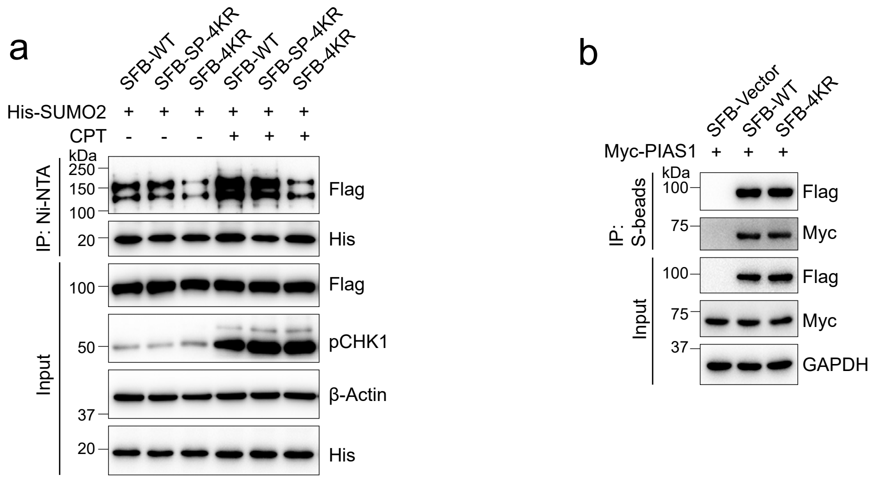

Supplementary Fig. 3 Screening for MRE11 SUMOylation sites.

a MRE11 with four software-predicted high-rank sites mutated into arginine (named SP-4KR) was constructed, and its SUMOylation level was analyzed. **b** 4KR mutation did not impair MRE11 interaction with PIAS1. Source data are provided as a Source Data file.

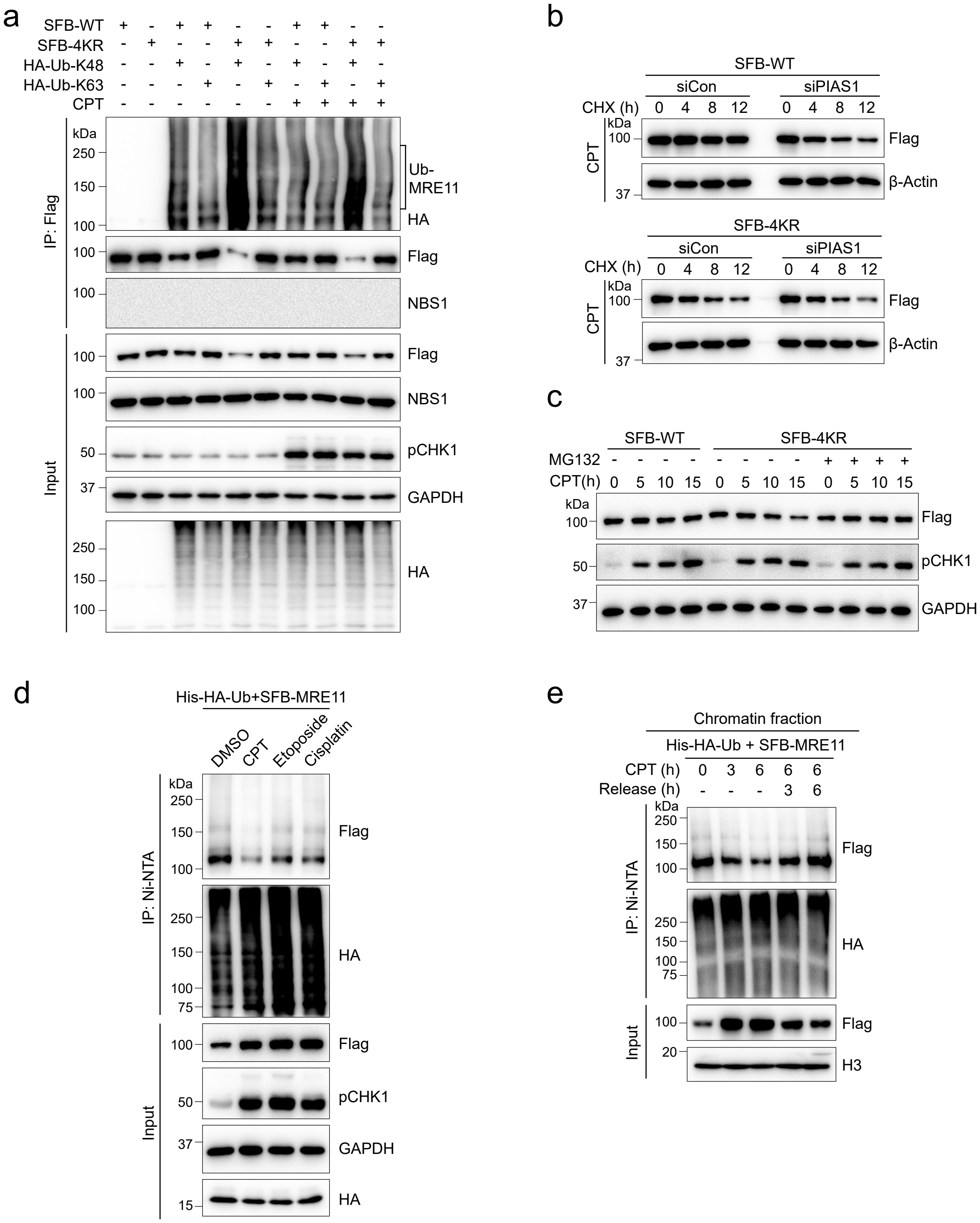
a
SFB-WT
SFB-4KR
HA-Ub-K48
HA-Ub-K63
CPT
kDa
250
150
100
Ub-MRE11
HA
IP: Flag
Flag
NBS1
Input
pCHK1
GAPDH
50
37
b
SFB-WT
siCon
siPIAS1
CHX (h)
0 4 8 12 0 4 8 12
CPT
β-Actin
SFB-4KR
c
MG132
CPT(h)
0 5 10 15 0 5 10 15 0 5 10 15
d
His-HA-Ub+SFB-MRE11
DMSO
Etoposide
Cisplatin
IP: Ni-NTA
75
15
e
Chromatin fraction
His-HA-Ub + SFB-MRE11
CPT (h)
0 3 6 6 6
Release (h)
3 6
20
H3

Supplementary Fig. 4 SUMOylation prevents MRE11 from ubiquitin-mediated degradation during DNA end resection.

a K48-linked ubiquitin chain dominates MRE11 ubiquitylation. HEK293T cells were transfected with the plasmids of ubiquitin K48- or K63-only lysine variants and other indicated plasmids, and cell lysates were subjected to Flag pull-down with SDS denaturing buffer and immunoblotting. **b** HeLa cells expressing SFB-WT and SFB-4KR were transfected with siPIAS1 and treated with 1 μM CPT for 2 h, followed by protein half-life analysis. **c** SFB-WT and SFB-4KR were transfected into HEK293T cells. Then, the cells were treated with 1 μM CPT and 1 μM MG132 as indicated, followed by immunoblotting. **d** HEK293T cells expressing His-HA-Ub and SFB-MRE11 were treated with DSB-inducing agents (1 μM CPT for 8 h, 100 μM etoposide for 6 h, and 1 μM cisplatin for 8 h). Then, MRE11 ubiquitylation was examined by Ni-NTA pull-down and immunoblotting. **e** HeLa cells transfected with His-HA-Ub and SFB-MRE11 were fractionated into soluble and chromatin fractions. The chromatin fractions were further subjected to Ni-NTA pull-down with denaturing buffer and immunoblotting. Source data are provided as a Source Data file.

a

b

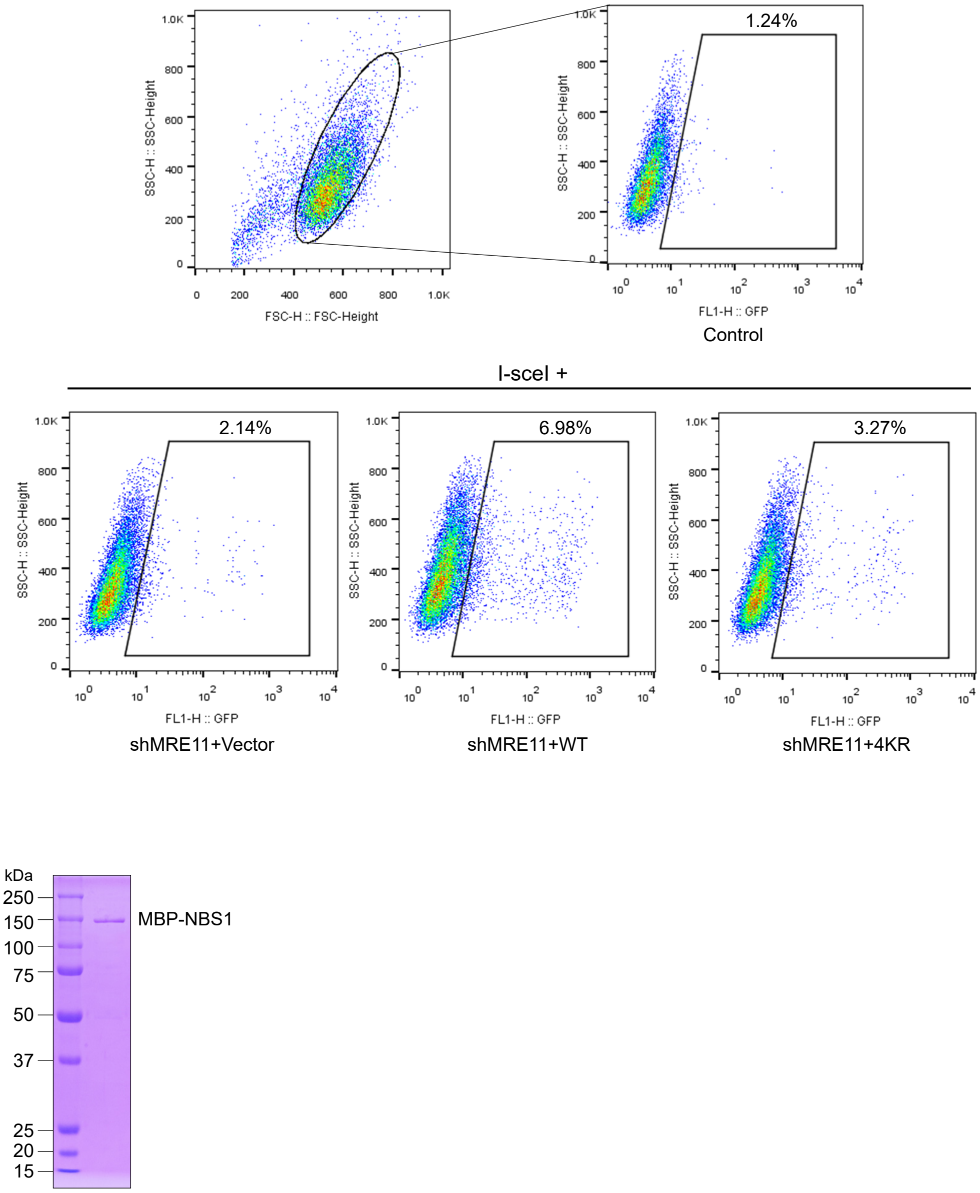

Supplementary Fig. 5 MRE11 SUMOylation promotes HR efficiency.

a Disruption of MRE11 SUMOylation caused defective HR. DR-GFP U2OS cells stably co-expressing SFB-Vector/SFB-WT/SFB-4KR and shMRE11 were infected with I-SceI lentivirus, then examined by flow cytometry. **b** MBP-tagged NBS1 protein was purified to near homogeneity, and then analyzed by SDS polyacrylamide gel electrophoresis and Coomassie blue staining. Source data are provided as a Source Data file.

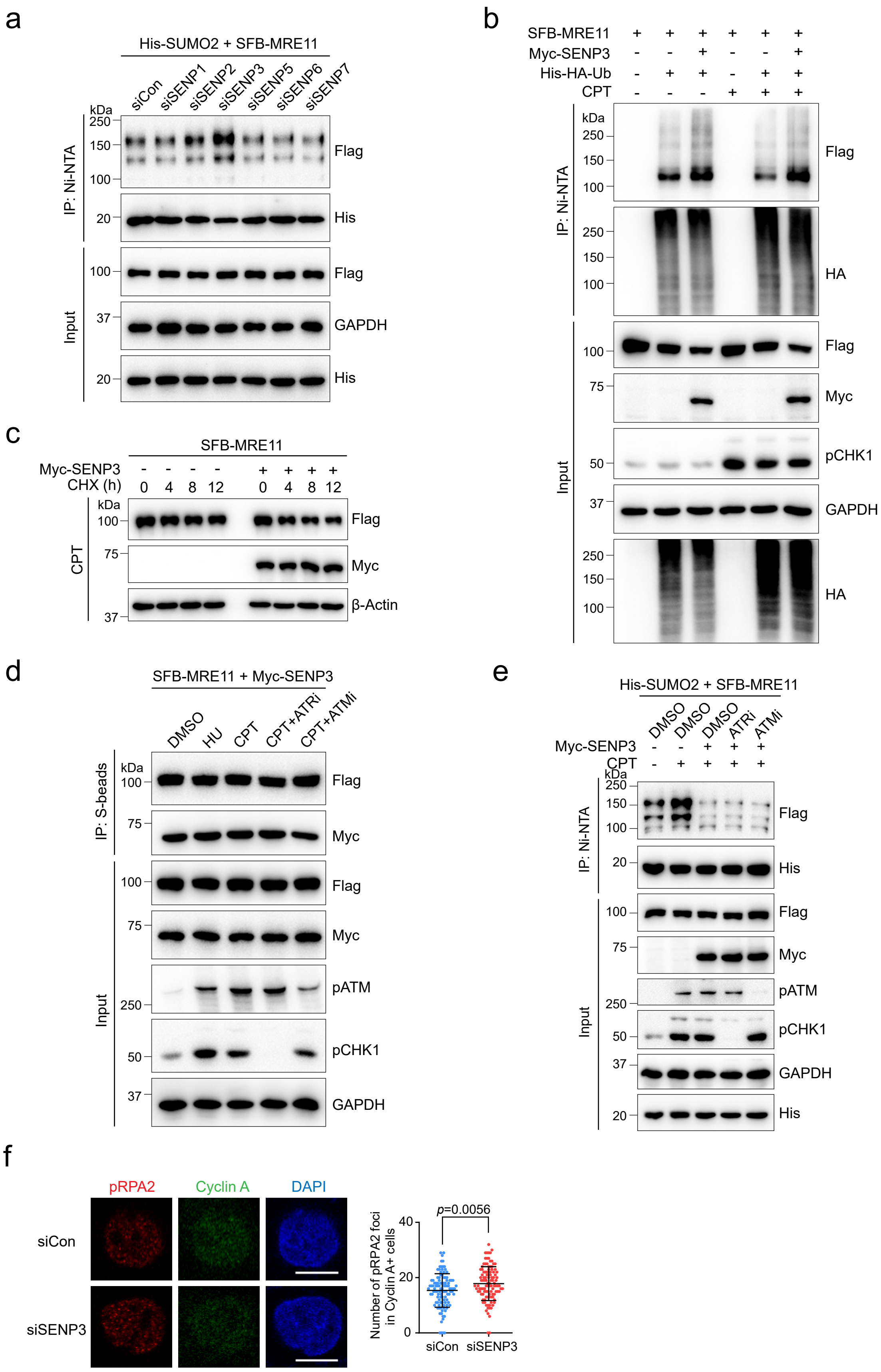
a
His-SUMO2 + SFB-MRE11
siCon
siSENP1
siSENP2
siSENP3
siSENP5
siSENP6
siSENP7
kDa
250
150
100
20
100
37
20
IP: Ni-NTA
Input
Flag
His
Flag
GAPDH
His
b
SFB-MRE11 + + + + + +
Myc-SENP3 - - + - - +
His-HA-Ub - + + - + +
CPT - - - + + +
kDa
250
150
100
IP: Ni-NTA
Input
Flag
HA
Flag
Myc
pCHK1
GAPDH
HA
75
50
37
c
SFB-MRE11
Myc-SENP3 - - - - + + + +
CHX (h) 0 4 8 12 0 4 8 12
CPT
Flag
Myc
β-Actin
d
SFB-MRE11 + Myc-SENP3
DMSO
HU
CPT
CPT+ATRi
CPT+ATMi
IP: S-beads
Input
Flag
Myc
Flag
Myc
pATM
pCHK1
GAPDH
e
His-SUMO2 + SFB-MRE11
DMSO
DMSO
DMSO
ATRi
ATMi
Myc-SENP3 - - + + +
CPT - + + + +
IP: Ni-NTA
Input
Flag
His
Flag
Myc
pATM
pCHK1
GAPDH
His
f
pRPA2
Cyclin A
DAPI
siCon
siSENP3
p=0.0056
Number of pRPA2 foci in Cyclin A+ cells
40
20
0
siCon
siSENP3

Supplementary Fig. 6 SENP3 deSUMOylates MRE11 mainly after DNA end resection.

a MRE11 SUMOylation was enhanced by SENP3 knockdown. HeLa cells cotransfected with SFB-MRE11, His-SUMO2 and the indicated siRNA were used to analyze MRE11 SUMOylation levels. **b** The ubiquitylation of MRE11 was enhanced by SENP3 overexpression in HeLa cells with His-HA-Ub. **c** SENP3 overexpression caused MRE11 half-life shortened under CPT treatment. **d** The interaction between Myc-SENP3 and SFB-MRE11 was not affected by HU (1 μM for 6 h), CPT (1 μM for 6 h), or the combination of CPT and ATRi (VE-821, 10 μM for 6 h) or ATMi (KU-55933, 10 μM for 6 h) treatment as indicated. **e** ATMi (KU-55933, 10 μM) and ATRi (VE-821, 10 μM) had no effect on MRE11 deSUMOylation by SENP3 after 1 μM CPT treatment for 8 h. **f** Knockdown of SENP3 caused excessive pRPA2 (S4/S8) foci formation in HeLa cells after 1 μM CPT treatment for 45 min. Scale bar, 10 μm. The data are presented as means ± SD (n = 108 cells). Source data are provided as a Source Data file.

Supplementary Table 1 Chemicals used in this study

Drugs	Company	Cat#
Camptothecin	HARVEYBIO	HZB0043
Etoposide	HARVEYBIO	HZB0098-25
Cisplatin	HARVEYBIO	C21384
Hydroxyurea	HARVEYBIO	HZB1502
MG132	LABLEAD	474790LB
Thymidine	HARVEYBIO	HZB1595-1
Olaparib	MedChemExpress	HY-10162
ATM inhibitor KU-55933	Selleck Chemicals	S1092
ATR inhibitor VE-821	TargetMol	T3032
Cycloheximide	HARVEYBIO	HZB0899-100

Supplementary Table 2 siRNA list

Target gene	Sequence (5’-3’)
MRE11 #1	GAGCAUAACUCCAUAAGUA
MRE11 #2	CCUGGUUGUUGUAGUAAGA
UBC9	GCAGAGGCCUACACGAUUU
PIAS1	GGAUCAUUCUAGAGCUUUA
SENP1	GGAAAUGGAGAAAGAAAUA
SENP2	CAUGCUGAAACUGGGUAAU
SENP3	ACUCCGUACCAAGGGUUAU
SENP5	GUCCACUGGUCUCUCAUUA
SENP6	GGACAAAUCUGCUCAGUGU
SENP7	GGCCAUGUAUUCUUAUACU
RNF4	GACAGAGACGUAUAUGUGA
Scrambled Control	UUCUCCGAACGUGUCACGU

Supplementary Table 3 Primers for qPCR

Gene	Primer (5'-3')
SENP1-F	CATTTCGCCTGACCATTACACGC
SENP1-R	CACACTTGGCAAGCCCTTCTCT
SENP2-F	CAGAGACGATGGTCGGAATCAG
SENP2-R	CCTCCTGAGTAAGCCATTGCTTC
SENP3-F	ATCCACCTGGAGGTGCATTGGT
SENP3-R	TCTTTACCGCCTCTGCCTGTAG
SENP5-F	GTCAGAAAGCCTCTCCAGTGGA
SENP5-R	CAAGGACTTCTTTTTCACTGAGTG
SENP6-F	GAGCATCAAAGGAAGTTGTGGGC
SENP6-R	GAAGATGGTGTGGTTTTCTCCAG
SENP7-F	TCTTTCCCTGCTGGTGTTGCTG
SENP7-R	CAACCGCTACTTTGCTTCTGCAG
GAPDH-F	ACCCACTCCTCCACCTTTGA
GAPDH-R	CTGTTGCTGTAGCCAAATTCGT
