# Supplementary material for: Crosstalk between SUMOylation and ubiquitylation controls DNA end resection by maintaining MRE11 homeostasis on chromatin
Source: Nat Commun. 2022 Sep 1;13:5133. doi: 10.1038/s41467-022-32920-x (PMC9436968; doi:10.1038/s41467-022-32920-x)
Supplement: Supplementary file 3 — Reporting Summary [file 41467_2022_32920_MOESM3_ESM.pdf]

nature portfolio

Corresponding author(s): Weibin Wang

Last updated by author(s): Aug 14, 2022

Reporting Summary

Nature Portfolio wishes to improve the reproducibility of the work that we publish. This form provides structure for consistency and transparency in reporting. For further information on Nature Portfolio policies, see our Editorial Policies and the Editorial Policy Checklist.

Statistics

For all statistical analyses, confirm that the following items are present in the figure legend, table legend, main text, or Methods section.

n/a	Confirmed	
☐	☒	The exact sample size (*n*) for each experimental group/condition, given as a discrete number and unit of measurement
☐	☒	A statement on whether measurements were taken from distinct samples or whether the same sample was measured repeatedly
☐	☒	The statistical test(s) used AND whether they are one- or two-sided<br>*Only common tests should be described solely by name; describe more complex techniques in the Methods section.*
☒	☐	A description of all covariates tested
☒	☐	A description of any assumptions or corrections, such as tests of normality and adjustment for multiple comparisons
☐	☒	A full description of the statistical parameters including central tendency (e.g. means) or other basic estimates (e.g. regression coefficient) AND variation (e.g. standard deviation) or associated estimates of uncertainty (e.g. confidence intervals)
☐	☒	For null hypothesis testing, the test statistic (e.g. *F*, *t*, *r*) with confidence intervals, effect sizes, degrees of freedom and *P* value noted<br>*Give P values as exact values whenever suitable.*
☒	☐	For Bayesian analysis, information on the choice of priors and Markov chain Monte Carlo settings
☒	☐	For hierarchical and complex designs, identification of the appropriate level for tests and full reporting of outcomes
☒	☐	Estimates of effect sizes (e.g. Cohen's *d*, Pearson's *r*), indicating how they were calculated

Our web collection on statistics for biologists contains articles on many of the points above.

Software and code

Policy information about availability of computer code

Data collection	Immunofluorescence images were acquired by ZEN (2.1, black); Electrophoretic mobility shift assay and nuclease assay images were acquired by Genesys (1.8.2); Immunoblotting images were acquired by Gelcap 5.6; qPCR data were collected by ABI Detector (StepOne Plus); Flow cytometric data were collected by CellQuest (3.3).<br>GPS-SUMO online software: http://sumosp.biocuckoo.org/online.php.<br>SUMOplot online software: https://www.abcepta.com.cn/tools.<br>JASSA online software: http://www.jassa.fr.
Data analysis	Immunofluorescence images were analyzed by ZEN (3.1, blue); Images of immunoblotting, electrophoretic mobility shift assay, and nuclease assay were analyzed by ImageJ (1.8.0). Flow cytometric data were analyzed by FlowJo (10.0.7). GraphPad Prism 8 software and SPSS 17.0 were used for statistical analyses.

For manuscripts utilizing custom algorithms or software that are central to the research but not yet described in published literature, software must be made available to editors and reviewers. We strongly encourage code deposition in a community repository (e.g. GitHub). See the Nature Portfolio guidelines for submitting code & software for further information.

Data

Policy information about availability of data

All manuscripts must include a data availability statement. This statement should provide the following information, where applicable:

- Accession codes, unique identifiers, or web links for publicly available datasets
- A description of any restrictions on data availability
- For clinical datasets or third party data, please ensure that the statement adheres to our policy

Data availability
All data supporting the findings of this study are available from the corresponding author. Source data are provided with this paper.
ClinVar and cBioPortal databases are publicly available.
ClinVar database: https://www.ncbi.nlm.nih.gov/clinvar/
cBioPortal database: http://www.cbioportal.org/

Field-specific reporting

Please select the one below that is the best fit for your research. If you are not sure, read the appropriate sections before making your selection.

☒ Life sciences ☐ Behavioural & social sciences ☐ Ecological, evolutionary & environmental sciences

For a reference copy of the document with all sections, see nature.com/documents/nr-reporting-summary-flat.pdf

Life sciences study design

All studies must disclose on these points even when the disclosure is negative.

Sample size	All sample sizes were based on experimental methods and our previous study (Bai et al. 2019. Mol Cell. PMID: 31353207).
Data exclusions	Data exclusions were not applied.
Replication	All experiments were independently replicated at least 3 times.
Randomization	All cells were randomly assigned to experimental groups.
Blinding	The investigators were blinded to experimental conditions before investigators finished data analysis.

Reporting for specific materials, systems and methods

We require information from authors about some types of materials, experimental systems and methods used in many studies. Here, indicate whether each material, system or method listed is relevant to your study. If you are not sure if a list item applies to your research, read the appropriate section before selecting a response.

Materials & experimental systems

n/a	Involved in the study	
☐	☒	Antibodies
☐	☒	Eukaryotic cell lines
☒	☐	Palaeontology and archaeology
☒	☐	Animals and other organisms
☒	☐	Human research participants
☒	☐	Clinical data
☒	☐	Dual use research of concern

Methods

n/a	Involved in the study	
☒	☐	ChIP-seq
☐	☒	Flow cytometry
☒	☐	MRI-based neuroimaging

Antibodies

Antibodies used

Immunoblotting (IB), Immunofluorescence (IF):
anti-Flag-HRP (Sigma-Aldrich, A8592, 1:2000) for IB;
anti-MBP-HRP (NEB, E8038, 1:3000) for IB;
anti-MRE11 (Proteintech, 10744-1-AP, 1:1000) for IB;
anti-MRE11 (Abcam, ab33125, 1:200) for IF;
anti-NBS1 (Proteintech, 55025-1-AP, 1:1000) for IB;
anti-RAD50 (ABclonal, A3078, 1:1000) for IB;
anti-RNF4 (Proteintech, 17810-1-AP, 1:1000) for IB;

anti-UBC9 (Proteintech, 51018-2-AP, 1:2000) for IB;
anti-PIAS1 (Proteintech, 23395-1-AP, 1:1000) for IB;
anti-phospho-RPA32 (S4/S8) (Abcam, ab87277) for IB (1:1000) and IF (1:500);
anti-RPA32 (Proteintech, 10412-1-AP, 1:1000) for IB;
anti-phospho-CHK1 (S345) (Cell Signaling Technology, 2348S, 1:1000) for IB;
anti-CHK1 (Santa Cruz, sc-8408, 1:1000) for IB;
anti-phospho-H2AX (S139) (Cell Signaling Technology, 9718, 1:1000) for IB;
anti-phospho-ATM (S1981) (Cell Signaling Technology, 5883S, 1:1000) for IB;
anti-phospho-NBS1 (S343) (Cell Signaling Technology, 3001S, 1:1000) for IB;
anti-RAD51 (Abcam, ab133534, 1:250) for IF;
anti-SUMO2/3 (Santa Cruz, sc32873, 1:500) for IB;
anti-SUMO2/3 (Abcam, ab81371, 1:500) for IB;
anti-SUMO1 (Santa Cruz, sc-5308, 1:200) for IB;
anti-Histone H3 (Biodragon, B1055, 1:2000) for IB;
anti-Cyclin A (Santa Cruz, sc-271682, 1:250) for IF;
anti-Myc (Santa Cruz, sc-40) for IB (1:1000) and IF (1:250);
anti-HA (BioLegend, 901503, 1:1000) for IB;
anti-His (Santa Cruz, sc-803, 1:1000) for IB;
anti-GAPDH (Sungene, KM9002, 1:5000) for IB;
anti-β-Actin (Sungene, KM9001, 1:5000) for IB.

Validation

anti-Flag-HRP for IB, https://www.sigmaaldrich.cn/CN/zh/product/sigma/a8592;
anti-MBP-HRP for IB, http://www.neb-china.com/pshow.asp?id=3613;
anti-MRE11 for IB, https://www.ptglab.com/Products/MRE11A-Antibody-10744-1-AP.htm;
anti-MRE11 for IF, https://www.abcam.cn/mre11-antibody-ab33125.html;
anti-NBS1 for IB, https://www.ptglab.com/products/NBN-Antibody-55025-1-AP.htm;
anti-RAD50 for IB, https://abclonal.com.cn/catalog/A3078;
anti-RNF4 for IB, https://www.ptglab.com/products/RNF4-Antibody-17810-1-AP.htm;
anti-UBC9 for IB, www.ptglab.com/products/UBC9-Antibody-51018-2-AP.htm;
anti-PIAS1 for IB, https://www.ptglab.com/products/PIAS1-Antibody-23395-1-AP.htm;
anti-phospho-RPA32 (S4/S8) for IB and IF, https://www.abcam.cn/rpa32rpa2-phospho-s4--s8-antibody-ab87277.html;
anti-RPA32 for IB, https://www.ptglab.com/products/RPA2-Antibody-10412-1-Ap.htm;
anti-phospho-CHK1 (S345) for IB, https://www.cellsignal.cn/products/primary-antibodies/phospho-chk1-ser345-133d3-rabbit-mab/2348?site-search-type=Products&N=4294956287&Ntt=2348s&fromPage=plp&_requestid=1841901;
anti-CHK1 for IB, https://www.scbt.com/p/chk1-antibody-g-4;
anti-phospho-H2AX (S139) for IB, https://www.cellsignal.cn/products/primary-antibodies/phospho-histone-h2a-x-ser139-20e3-rabbit-mab/9718?site-search-type=Products&N=4294956287&Ntt=h2ax&fromPage=plp;
anti-phospho-ATM (S1981) for IB, https://www.cellsignal.cn/products/primary-antibodies/phospho-atm-ser1981-d6h9-rabbit-mab/5883?site-search-type=Products&N=4294956287&Ntt=5883s&fromPage=plp&_requestid=1842322;
anti-phospho-NBS1 (S343) for IB, https://www.cellsignal.cn/products/primary-antibodies/phospho-p95-nbs1-ser343-antibody/3001?_=1646978164176&Ntt=3001s&tahead=true;
anti-RAD51 for IF, https://www.abcam.cn/rad51-antibody-epr40303-ab133534.html;
anti-SUMO2/3 for IB, https://www.scbt.com/p/sumo-2-3-antibody-fl-103?requestFrom=search;
anti-SUMO2/3(abcam) for IB, https://www.abcam.cn/sumo-2--sumo-3-antibody-8a2-ab81371.html;
anti-SUMO1 for IB, https://www.scbt.com/zh/p/sumo-1-antibody-d-11?requestFrom=search;
anti-Histone H3 for IB, https://www.biodragon.cn/nckt/73664.html;
anti-Cyclin A for IF, https://www.scbt.com/zh/p/cyclin-a-antibody-b-8?requestFrom=search;
anti-Myc for IB and IF, https://www.scbt.com/zh/p/c-myc-antibody-9e10
anti-HA for IB, https://www.biolegend.com/en-us/products/purified-anti-ha-11-epitope-tag-antibody-11374;
anti-His for IB, https://www.scbt.com/p/his-probe-antibody-h-15?requestFrom=search;
anti-GAPDH for IB, http://www.sungenebiotech.com/index.php?m=Product&a=product_xq&catid=2&proid=53&prid=290&pid=720&id=1557;
anti-β-Actin for IB, http://www.sungenebiotech.com/index.php?m=Product&a=product_xq&catid=2&proid=53&prid=289&pid=717&id=1548.

Eukaryotic cell lines

Policy information about cell lines

Cell line source(s)	HEK293T, HeLa and U2OS cell lines were purchased from ATCC.
Authentication	Cell lines authentication was identified by short tandem repeat profiling.
Mycoplasma contamination	All cell lines were confirmed without mycoplasma contamination.
Commonly misidentified lines (See ICLAC register)	No commonly misidentified cell lines were used in this study.

Flow Cytometry

Plots

Confirm that:

☒ The axis labels state the marker and fluorochrome used (e.g. CD4-FITC).

☒ The axis scales are clearly visible. Include numbers along axes only for bottom left plot of group (a 'group' is an analysis of identical markers).

☒ All plots are contour plots with outliers or pseudocolor plots.

☒ A numerical value for number of cells or percentage (with statistics) is provided.

Methodology

Sample preparation	For HR assay, after 48 h I-sceI lentivirus transfection, cells were trypsinized and collected for flow cytometric analysis.
Instrument	BD FACS Calibur.
Software	FACS data were collected and analyzed by CellQuest and FlowJo.
Cell population abundance	No sorting was performed.
Gating strategy	Control group and I-sceI group were gated based on FSC and SSC for appropriate gates. Then, GFP-positive cells were gated in 488 nm channel.

☒ Tick this box to confirm that a figure exemplifying the gating strategy is provided in the Supplementary Information.
